# Effects of endophytes on early growth and ascorbate metabolism in *Brassica napus*

**DOI:** 10.3389/fpls.2024.1480387

**Published:** 2024-12-12

**Authors:** Susan Jones-Held, James F. White

**Affiliations:** Department of Plant Biology, Rutgers University, New Brunswick, NJ, United States

**Keywords:** endophytes, canola, seedling growth, ascorbate oxidation, priming, ROS (reactive oxygen species)

## Abstract

Understanding the early interactions between plants and endophytes will contribute to a more systematic approach to enhancing endophyte-mediated effects on plant growth and environmental stress resistance. This study examined very early growth and ascorbate metabolism after seed treatment of *Brassica napus* with three different endophytes. The three endophytes used were *Bacillus amyloliquefaciens* pb1(Bapb1), *Micrococcus luteus* (Ml) and *Pseudomonas fluorescens* SLB4 (SLB4). Seeds of *Brassica napus* cv. trophy were surface sterilized and plated on 1/2 MS Basal salts (pH 5.7 -5.8) + 0.8% agarose. Under sterile conditions, endophyte suspensions or sterile distilled water (controls) were applied to plated seeds. After two days, all plates were scanned to produce digital images for subsequent growth analysis. Then, seedlings were gently removed from the plates and placed in sterile microfuge tubes. For biochemical analyses, extracts were prepared from samples and assayed spectrophotometrically. We detected slight changes in seedling root tip and/or primary root growth with Bapb1 and Ml. Seedlings treated with SLB4 exhibited significantly increased primary root and root tip length after two days of growth. Ascorbate oxidation, however, was the primary significant change common to all endophyte-treated seedlings. In relation to ascorbate oxidation, soluble ascorbate oxidase (AO) was slightly reduced in Bapb1 and Ml-treated seedlings, whereas ionically-bound AO was reduced in Bapb1 and SLB4-treated seedlings. Total AO activity was significantly reduced in Bapb1-treated seedlings. There were no differences in cytosolic APX activity or glutathione levels between endophyte-treated seedlings and controls. Like pathogens, endophytes can trigger an oxidative burst in the plant. A level of ascorbate oxidation seems required to propagate ROS as signaling molecules as part of the plant immune response. The slight to moderate reductions in plant AO activity that we found mimic the inhibitory effects of pathogens on AO activity, but there was still a level of AO activity that may have been sufficient for the apoplastic ascorbate oxidation required for subsequent ROS signaling. Other studies have suggested that endophytes may elicit a more moderate plant immune response relative to pathogens to facilitate colonization. The AO, APX, and glutathione results would be consistent with a moderate plant immune response to endophytes.

## 1 Introduction

Numerous studies have demonstrated that bacterial endophytes may benefit subsequent plant growth and provide resilience to environmental stress. These studies have included a variety of plants and endophytes (Irizarry and White, 2017; Verma et al., 2018; Fan et al., 2020; Mellidou et al., 2021). Enhancing plant growth, stress tolerance, and overall productivity using beneficial bacteria may be important to expanding sustainable agriculture. Furthermore, understanding plant-endophyte interactions may help mitigate climate change effects on agrosystems and natural ecosystems (Compant et al., 2021; de Vries et al., 2020).

The interactions between plant and endophyte(s) are complex. Early work recognized the significance of understanding the initial interactions between the plant and endophyte in establishing a relationship that may improve plant growth and stress tolerance (Hallmann et al., 1997; Preston, 2004). More recent reviews emphasize the need for further research to understand the dynamics of these early interactions (Pinski et al., 2019; Compant et al., 2021; de Vries et al., 2020). Because of the significance of the initial plant-endophyte interaction, we focused our study on early seedling growth and response to endophytes. We chose *Brassica napus* for several reasons: 1) canola is an economically important crop (Card et al., 2015; Rybakova et al., 2017); 2) seed germination and measurable growth occur in a short time; and 3) as a member of the Brassicaceae, canola is related to *Arabidopsis thaliana* which has been intensively studied for decades as the first model plant. There is today a well-documented mechanistic understanding of the interaction of *Arabidopsis* with both pathogenic and nonpathogenic bacteria, including to a lesser extent endophytic bacteria (Millet et al., 2010; Zamioudis et al., 2013; Ortiz-Castro et al., 2014; Asari et al., 2017). Thus, this knowledge may provide insight into molecular, biochemical, and/or physiological responses of canola to endophytic bacteria.

Three different endophytes were used in this study. Each endophyte (*Bacillus amyloliquefaciens* pb1, *Pseudomonas fluorescens* SLB4, and *Micrococcus luteus*) was isolated from a different plant host. Members of these three genera are widely present in the seeds of diverse plant species (Taulé et al., 2021). Two of the endophytes, *Bacillus amyloliquefaciens* pb1 and *Pseudomonas fluorescens* SLB4, have been shown to affect plant growth and response to abiotic and biotic factors (Irizarry and White, 2017; White et al., 2018b; Verma et al., 2018). A microscopic examination indicated that *Micrococcus luteus* may also affect plant growth (White et al., 2018b).

One of the initial responses of plants when exposed to endophytes is an oxidative burst producing reactive oxygen species (ROS) such as superoxide anion and hydrogen peroxide (Zhou et al., 2016; Gerna et al., 2020). The endophyte and plant must control ROS produced to prevent cellular damage. However, at the same time, generated ROS may be involved in signaling between the endophyte and plant and within the plant or endophyte itself (Pan et al., 2019; Gerna et al., 2020). In this study, we analyzed the nonenzymatic antioxidants ascorbate and glutathione as part of the initial plant response to exposure to the endophyte and ROS production. Ascorbate and glutathione are significant plant antioxidants (Foyer and Noctor, 2011; Foyer and Kunert, 2024). Involvement of enzymatic and nonenzymatic antioxidants in plant-endophyte interactions has primarily focused on plants exposed to environmental stress rather than the actual plant-endophyte interaction itself under normal conditions. In our studies, we also examined the ascorbate utilizing enzymes, ascorbate oxidase and cytosolic ascorbate peroxidase, because of their essential roles in modulating ascorbate and ROS (De Tullio et al., 2013; Foyer et al., 2020; Kaur et al., 2021). Recently, the importance of ascorbate oxidase and ascorbate peroxidase during the early stages of plant-pathogen interactions has been demonstrated (Hu et al., 2022; Hong et al., 2023).

## 2 Materials and methods

### 2.1 Bacterial cultures and preparation for seed inoculation

Bacterial cultures were routinely maintained on LB (Luria-Bertani) plates at room temperature. Three different bacterial species were used: *Bacillus amyloliquefaciens* pb1 (GenBank Accession # KX622565), *Pseudomonas fluorescens* SLB4 (GenBank Accession # KX665565), *Micrococcus luteus* isolated from *Solanum esculentum* cv. Beefsteak (J. F. White, pers. comm.).

Each bacterial culture was used to inoculate and establish an overnight broth culture (LB) on a rotary shaker (100 rpm) at room temperature. Aliquots from each overnight culture were pelleted and washed 2X with sterile distilled water. After the second wash, the pellets were resuspended in sterile distilled water. The OD_600nm_ of each bacterial suspension was recorded. A portion of the washed and resuspended bacteria was serially diluted and plated. For seed inoculation, *Bacillus amyloliquefaciens* and *Micrococcus luteus* were each used at a concentration of 10^7^cfu/mL. *Pseudomonas fluorescens* was used at a concentration of 10^9^cfu/mL. The cell concentrations reflected the extent of overnight growth, and concentrations were not adjusted to make them uniform since different species were used.

### 2.2 Seed sterilization, treatment, and growth

Seeds of *Brassica napus* cv. trophy were added to sterile 15 mL conical tubes at a final volume of 1 mL of seeds per tube. Five mL of 10% Chlorox + 0.1% Tween 20 was added to each tube and shaken for 5 min. The liquid was removed and replaced with 5 mL of 95% ethanol. The tubes were shaken for 1 min. The seeds were thoroughly washed 3X with sterile distilled water and dispensed on plates that contained 1/2 MS Basal salts (pH 5.7 -5.8) + 0.8% agarose. The seeds were positioned and spaced on each plate in a single row, with approximately nine seeds comprising a row. Plates with sown seeds were placed at 4°C for two days before bacterial treatment. A preliminary experiment indicated that a two-day cold treatment resulted in more uniform seed germination and growth.

Under sterile conditions, 5 µL of washed bacterial suspension was applied to each seed on a plate. For control plates, 5 µL of sterile distilled water was applied to each seed on a plate. Replicate plates (2-3) were used for each application (bacterial- treated and control) and for each set of experiments. The plates were laid flat for 1 hour to allow further absorption of the 5 µL droplets applied. Subsequently, all plates were placed vertically under fluorescent lights at ambient temperature. The lights were on a 16-hour light/8-hour dark cycle at 92 µmol m^-2^ s^-1^. Each of the three bacterial species applied to seeds of *Brassica napus* had no effect on total seed germination compared to controls after two days of incubation (data not shown). After two days, all plates were scanned using a flatbed scanner to produce digital images for subsequent growth analysis. Growth measurements were done using ImageJ (Schneider et al., 2012). Individual seedlings were gently removed from the plates and placed in sterile microfuge tubes (3-4 seedlings per tube). The fresh weight of each sample was recorded. All samples were stored at -80°C until analyses.

### 2.3 Biochemical assays

All assays were done at room temperature using a Genesys 10S UV-Vis spectrophotometer.

#### 2.3.1 Ascorbate and glutathione

Seedling extracts were prepared using 1% metaphosphoric acid + 1 mM EDTA. The extract volume used was 1 mL/100 mg FW. Seedlings were ground using microfuge pestles, and homogenates were centrifuged at 14,000 rpm for 10 min. The supernatant of each sample was stored in aliquots at -80°C until assay.

Ascorbate and dehydroascorbate (DHA) were determined according to Luwe et al. (1993) with modification. Each 500 µL assay consisted of 50 µL of extract and 450 µL of 100 mM sodium phosphate buffer (pH 6.8). For ascorbate oxidation, 1 unit of ascorbate oxidase (from *Cucurbita sp.*) was used for each 500 µL assay. A second 500 µL assay was used for the reduction of DHA by addition of dithiothreitol to a final concentration of 0.2 mM. Absorbance was monitored at 265nm, and an extinction coefficient of 14.3 mM^-1^cm^-1^ was used.

Analysis of glutathione (GSH) and oxidized glutathione (GSSG) was based on the enzymatic recycling assay with modification (Griffith, 1980). Glutathione reductase, 5,5’-dithiobis-2-nitrobenzoic acid (DTNB), and NADPH were prepared in the assay buffer (100 mM sodium phosphate (pH 7.5) and 5 mM EDTA). For total GSH, each 500 µL assay consisted of 250 or 275 µL of assay buffer, 100 µL 6 mM DTNB, 25 or 50 µl sample extract or GSH standard, and 50 µL glutathione reductase (1 unit from baker’s yeast). Each reaction was initiated by adding 50 µL of 10 mM NADPH. The increase in absorbance at 412nm was monitored at 30 s intervals for 5 min. A second set of samples or standards were incubated with 2-vinylpyridine (0.84% final concentration) for 1 hour at 25°C and then used to measure GSSG as described for the total GTH assay. The difference between total GTH and GSSG was used to determine reduced glutathione.

Ascorbate oxidase and glutathione reductase were obtained from Sigma-Aldrich.

#### 2.3.2 Ascorbate oxidase and ascorbate peroxidase

Soluble ascorbate oxidase and cytosolic ascorbate peroxidase were assayed according to Wu et al. (2021) with minor modifications. Extracts from frozen plant samples were prepared as described above except that the homogenizing solution was 100 mM sodium phosphate buffer, pH 6.5. After grinding, samples were centrifuged at 14,000 rpm for 5 minutes. The sample supernatants were aliquoted into microfuge tubes.

The pellets remaining after homogenization of frozen plant tissue were resuspended in 100 mM sodium phosphate buffer, pH 6.5, containing 1 M NaCl at the same volume as the initial extracts. The pellets were vigorously vortexed for 20 s, followed by centrifugation at 14,000 rpm for 5 min., and transfer of the supernatants to fresh tubes. These extracts represented the ionically-bound ascorbate oxidase. All extracts were stored at -80°C until assay.

Soluble and ionically-bound ascorbate oxidase activity was determined by monitoring the decrease in absorbance at 265 nm over 5 min. in an assay buffer of 100 mM sodium phosphate (pH 5.8), 0.5 mM EDTA, and 50 µM ascorbate. The reactions were initiated by addition of sample extracts. The slope of the linear portion of the change in absorbance over time was measured; an extinction coefficient of 14 mM^-1^cm^-1^ was used to calculate activity.

Ascorbate peroxidase (cytosolic) was assayed in 100 mM sodium phosphate (pH 6.8) and 0.12 mM ascorbate; the reaction was initiated by adding 0.03% H_2_O_2_ (v/v). Two or three replicates were performed for each sample. Cytosolic ascorbate peroxidase activity was determined by monitoring the decrease in absorbance at 290 nm for 2 min. at room temperature. The slope of the linear portion of the change in absorbance over time was determined, and an extinction coefficient of 2.8 mM^-1^cm^-1^ was used to determine ascorbate peroxidase activity.

## 3 Results and discussion

### 3.1 Growth

Studies on the effects of endophytes on plant growth have contributed to identifying some of the complex signals between endophytes and plants. When comparing studies, however, the following factors must be considered: bacterial strains used, mode of bacterial inoculation, amount of bacterial inoculum (Ramírez and Kloepper, 2010), timeframe of treatment with bacteria or co-cultivation, procedure used for surface sterilization of seeds, plant species/cultivars used (Rybakova et al., 2017), and stage of plant growth used for analysis. Despite the variability of these factors, some of the identified chemical cues commonly related to growth effects are hormones. Hormonal effects on plant growth can be subtle, variable, and time dependent.

Hypocotyl and primary root growth in our experiments were unaffected by treatment of *Brassica napus* seeds with *Bacillus amyloliquefaciens* pb1 (Bapb1) after two days of incubation (**Table 1**). Root tip length was slightly increased in Bapb1 treated seedlings, but the increase was not statistically significant. Irizarry and White (2017) reported increased germination and seedling radicle length of *Gossypium hirsutum* (cotton) using Bapb1. This specific bacterial strain was initially isolated from *Thespesia populnea*, a member of the Malvaceae family which includes cotton (Irizarry and White, 2017). The taxonomic relationship between the plant source of the endophyte and cotton may have enhanced endophyte colonization and growth of *Gossypium hirsutum* relative to *Brassica napus*. This explanation would be consistent with another study where several different strains of *Bacillus amyloliquefaciens* isolated from *Gossypium barbadense* (Sea Island cotton) had very low rates of colonization of *Brassica napus* seedling roots (Reva et al., 2004). In contrast, *Bacillus amyloliquefaciens* strain UCMB5113 isolated from soil and two other isolates from *Arabidopsis thaliana* effectively colonized *Brassica napus* (Reva et al., 2004). The authors suggested the variability in colonization may be due to endophyte-host plant specificity. A subsequent study that used two of the same *Bacillus amyloliquefaciens* strains isolated from cotton and UCMB5113 as Reva et al. (2004) found no effects on germination rates or overall growth of *Brassica napus* by these strains (Danielsson et al., 2007). The only growth parameter affected was that each endophytic strain increased the number of plants that survived until seed set compared to control plants (Danielsson et al., 2007). In contrast, Asari et al. (2017) found that the length of primary roots of 11-day-old *Arabidopsis thaliana* Col-0 seedlings were significantly reduced when treated with *Bacillus amyloliquefaciens* UCMB5113. An increase in the number of lateral roots and root hairs accompanied the reduction of primary root length. These growth effects were not only dependent on the amount of inoculum used but were also time dependent. Only after three days of co-cultivation of the endophyte with the seedling was there a gradual reduction in primary root length and an increase in lateral root production compared to untreated plants. Using various experimental approaches, Asari et al. (2017) determined that UCMB5113-treated seedlings had altered levels of endogenous plant hormones such as auxin, cytokinins, gibberellins, and brassinosteroids compared to control seedlings. Interestingly, the authors reported that UCMB5113 in culture could produce IAA and cytokinins. Irizarry and White (2017) reported no tryptophan-dependent IAA production with Bapb1 cultures. However, their analysis was limited, and the results were qualitative.

**Table 1:** Growth parameters of two-day old *Brassica napus* seedlings treated with *Bacillus amyloliquefaciens* pb1 or untreated controls (n = 22-46).

Structure	Control Mean ± (SEM) in mm	Treated Mean ± (SEM) in mm
Hypocotyl length	4.5 (0.3)	4.7 (0.2)
Primary root length	12.3 (0.6)	12.1 (0.6)
Root tip length	2.1 (0.2)	2.6 (0.2)

Root tip length was determined by measuring from the tip of the root to the point where root hairs first emerge on the primary root. The sample size reflects the combined results of two independent experiments, with a total of 5-6 replicate plates for each endophyte treatment and control.

With Chinese cabbage, a member of the Brassicaceae, Ramírez and Kloepper (2010) found that seven-day-old seedlings that had been inoculated as seed with *Bacillus amyloliquefaciens* (FZB45) and grown under gnotobiotic conditions had significantly longer root lengths when compared to controls. This growth effect was inoculum dose dependent. A lower amount of bacterial inoculum significantly increased root length, whereas a higher amount did not. This response may be due to the effects of indole acetic acid (IAA) on root elongation (Ramírez and Kloepper, 2010). This strain (FZB45) was capable of producing IAA *in vitro*.

Studies using *Brassica napus* and *Micrococcus luteus* have primarily examined the effects of antimicrobial peptides produced by *B. napus* on *M. luteus* as a Gram-positive test organism (Cao et al., 2015; Ke et al., 2015). Recently, García-Cárdenas et al. (2022) examined the effects of a strain of *Micrococcus luteus* originally isolated from maize roots on the growth of *Arabidopsis thaliana* Col-0 seedlings. Similar to findings reported by Asari et al. (2017) (see above), the growth effects were time dependent. The primary root length was reduced after two days of co-cultivation, but the reduction in root length was only statistically significant after three days of growth under axenic conditions (García-Cárdenas et al., 2022). Their results are consistent with the minor reductions in hypocotyl length, primary root, and root tip length in *Brassica napus* treated with *Micrococcus luteus* that we observed after a two-day growth period and using a different method of bacterial application (**Table 2**). However, the results were not statistically significant.

**Table 2:** Growth parameters of two-day old *Brassica napus* seedlings treated with *Micrococcus luteus* or untreated controls (n = 39-57).

Structure	Control Mean ± (SEM) in mm	Treated Mean ± (SEM) in mm
Hypocotyl length	7.0 (0.2)	6.6 (0.3)
Primary root length	15.3 (0.7)	13.8 (0.7)
Root tip length	3.3 (0.2)	2.8 (0.2)

Root tip length was determined by measuring from the tip of the root to the point where root hairs first emerge on the primary root. The sample size reflects the combined results of two independent experiments, with a total of 5-6 replicate plates for each endophyte treatment and control.

To determine if the effect of *Micrococcus luteus* (Ml) on root growth may involve auxin, García-Cárdenas et al. (2022) used a transgenic *Arabidopsis* line that contained an auxin-inducible construct. When this line was co-cultivated with Ml, the construct was strongly expressed in the primary root and root tip of seedlings in physical contact with the bacterial inoculum compared to the controls. Further examination indicated that root contact with the bacteria significantly reduced the meristematic region of the root tip and the cortical cell length in the elongation region (García-Cárdenas et al., 2022). Reductions in the meristematic and/or elongation zones may account for the slight reduction in root growth that we found in our studies. It is unknown if the Ml strain we used produces IAA.

In contrast to the other two endophytic species used in our studies, *Pseudomonas fluorescens* SLB4 significantly enhanced root tip and primary root length in *Brassica napus* during the two-day incubation period (**Table 3**). Consistent with these results, Verma et al. (2018) found that SLB4 enhanced the root and shoot length of seven-day-old rice and Bermuda grass seedlings. Also, White et al. (2018a) reported that SLB4 increased the root and shoot length of Poa annua over 37 days compared to uninoculated plants. ROS and/or auxin signaling, or mobilization of inorganic and organic nutrients were suggested as possible factors affecting seedling growth by interaction with SLB4 (Verma et al., 2018).

**Table 3:** Growth parameters of two-day old *Brassica napus* seedlings treated with *Pseudomonas fluorescens* SLB4 or untreated controls (n = 36-48).

Structure	Control Mean ± (SEM) in mm	Treated Mean ± (SEM) in mm
Hypocotyl length	6.7 (0.3)	7.3 (0.3)
Primary root length	15.2 (0.5)	17.2 (0.7)*
Root tip length	2.9 (0.2)	3.6 (0.2)**

Root tip length was determined by measuring from the tip of the root to the point where root hairs first emerge on the primary root. The sample size reflects the combined results of two independent experiments, with a total of 5-6 replicate plates for each endophyte treatment and control. p-values are indicated when significantly different and were determined using an unpaired two-tailed t-test (*0.03; **0.006).

Another factor relevant to understanding the early growth effects of *Pseudomonas fluorescens* on *Brassica napus* is bacterial ACC (1-aminocyclopropane-1-carboxylic acid) deaminase activity. In two reviews, Glick and colleagues proposed that bacterial ACC deaminase activity is part of the key to PGPR (plant growth promoting rhizobacteria) function in plant growth and in plant responses to abiotic and biotic stresses (Glick, 2014; Gamalero et al., 2023). This bacterial enzyme catalyzes the breakdown of the ethylene precursor, ACC, to ammonia and alpha-ketobutyrate. The result is reduced amounts of ACC, which, in turn, lowers the levels of ethylene that may inhibit plant growth and responses to environmental stress. This model is dependent on the plant exuding ACC and the endophyte degrading ACC.

In support of the role of ACC deaminase activity on plant growth, Wang et al. (2000) transformed a *Pseudomonas fluorescens* strain that cannot use ACC as a nitrogen source with an ACC deaminase gene and its regulatory region. The transformants then grew on media containing ACC as the sole nitrogen source, evidence that they now had ACC deaminase activity. Transformed *Pseudomonas fluorescens* significantly increased the root length of four-day-old canola seedlings compared to the non-transformed strain. In another study, Penrose et al. (2001) found that canola seeds treated with different bacteria with ACC deaminase activity increased root length of 4.5-day-old canola seedlings. Upon further analysis, the seedling roots had significantly lower ACC levels than the controls. Similarly, seedlings from seeds treated with the ethylene inhibitor aminoethoxyvinylglycine (AVG) had increased root length and reduced ACC compared to controls (Penrose et al., 2001).

In an extensive study, Hynes et al. (2008) identified endophytic isolates that phylogenetically clustered as *Pseudomonas fluorescens* from peas, lentils, and chickpeas. Five of these isolates exhibited ACC deaminase activity but showed no indole production. All five isolates significantly increased the root length of seven-day-old *Brassica napus* seedlings compared to uninoculated controls under gnotobiotic conditions. Four other isolates, all negative for ACC deaminase activity, did not enhance root elongation in canola. However, one remaining isolate that exhibited ACC deaminase activity had no effect on growth on canola. Overall, these studies support the role of bacterial ACC deaminase, and (by logical extension) ethylene, in regulating plant growth in certain endophyte-plant interactions.

Other work using different strains of *Pseudomonas fluorescens* (Pf) and different varieties or cultivars of *Brassica napus* reported increases in seedling root length when treated with Pf (Sheng et al., 2008; Pallai et al., 2012; Chlebek et al., 2020). Among these studies, however, the Pf strains varied as to whether ACC deaminase was present or not detected. These results are only partially consistent with the role of ACC deaminase in affecting plant growth. Further research is needed to determine whether ACC deaminase activity is present in *Pseudomonas fluorescens* SLB4.

### 3.2 Ascorbate metabolism

**Figure 1:**
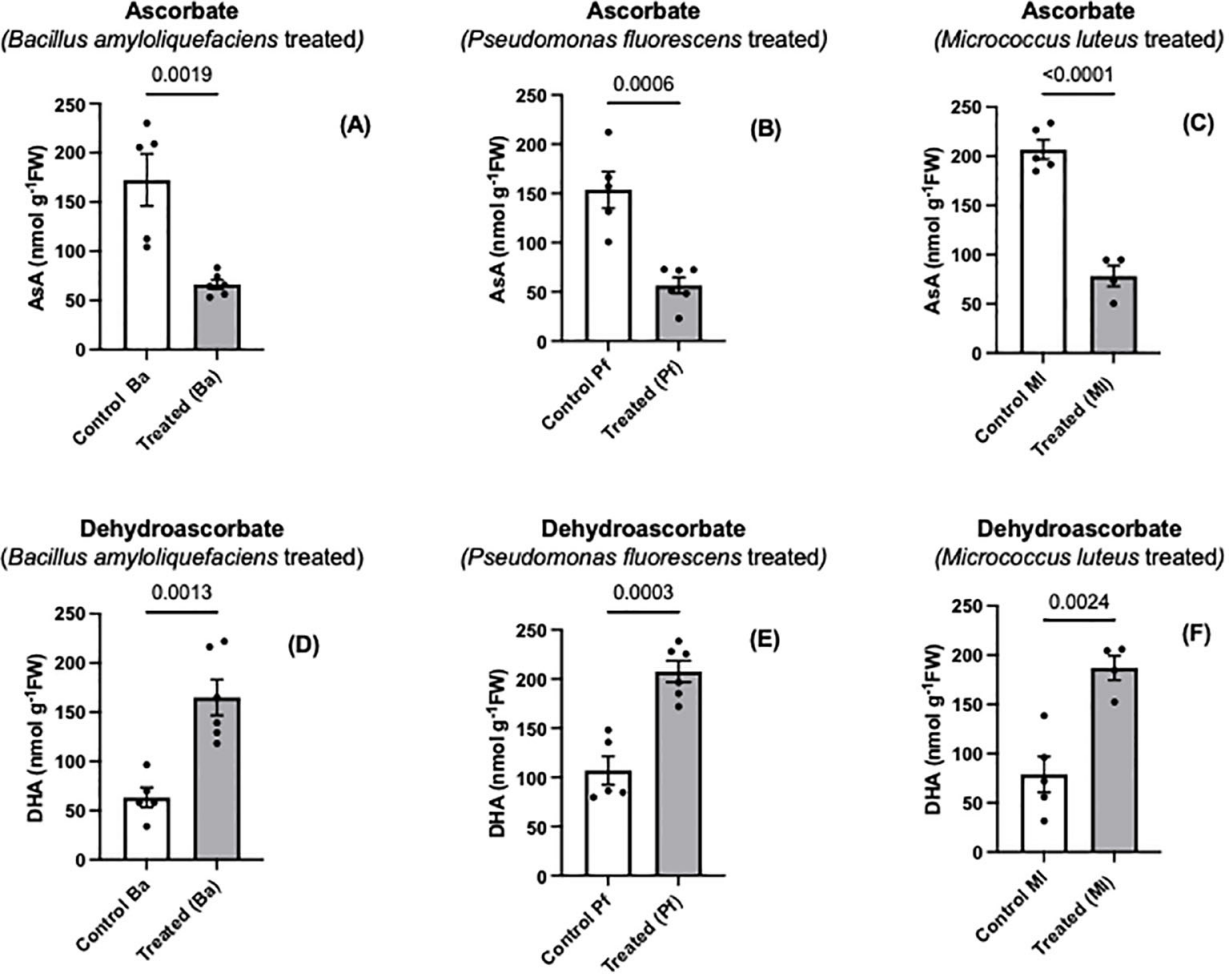
Ascorbate **(A-C)** and dehydroascorbate **(D-F)** levels in extracts prepared from two-day old *Brassica napus* seedlings treated with different endophytes or untreated controls. Mean ± SEM are indicated (n = 4-6). p-values are indicated when significantly different and were determined using an unpaired two-tailed t-test.

**Figure 2:**
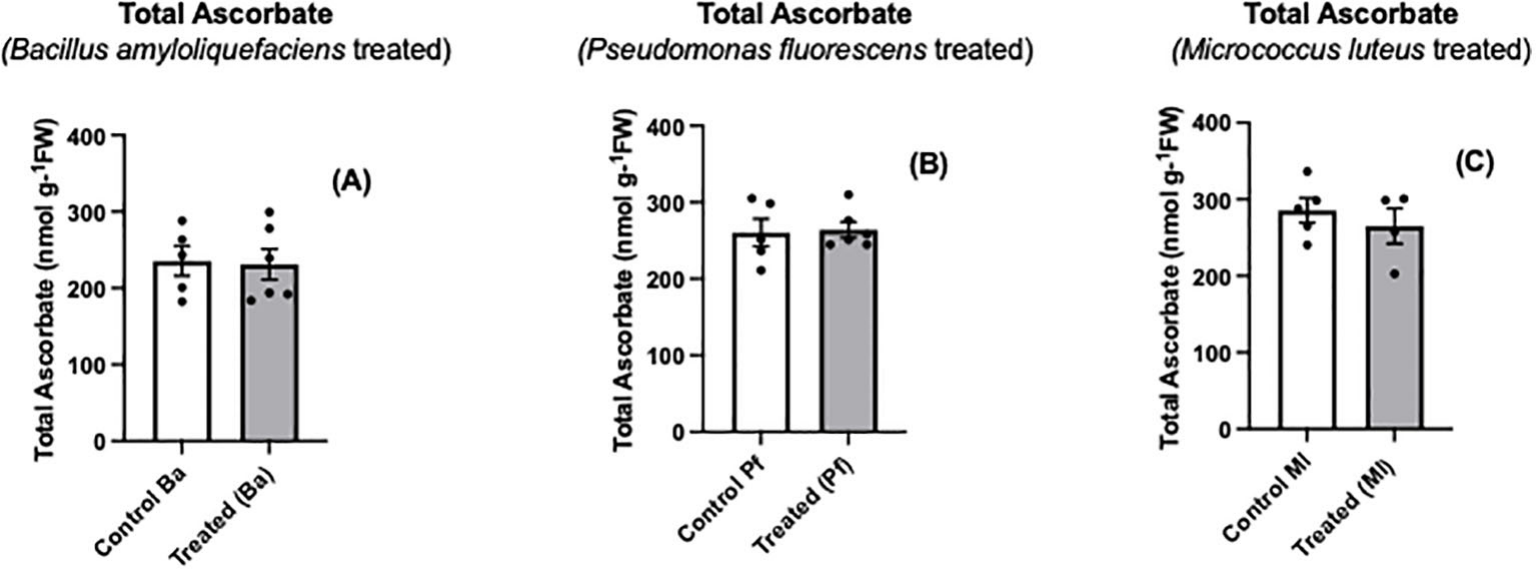
Total ascorbate levels **(A-C)** in extracts prepared from two-day old *Brassica napus* seedlings treated with different endophytes or untreated controls. Mean ± SEM are indicated (n = 4-6).

Ascorbate is one of the major nonenzymatic antioxidants of plant cells (Smirnoff, 2000; Foyer et al., 2020; Foyer and Kunert, 2024). When *Brassica napus* seeds were treated with three different endophytes, a significant oxidation of seedling ascorbate occurred, and the extent of ascorbate oxidation was similar regardless of the bacterial species used (**Figures 1A–F**). In contrast, there was no change in the total amount of ascorbate (ascorbate + dehydroascorbate) between endophyte-treated seedlings and controls (**Figures 2A–C**). Similar to the early stages of plant-pathogen interactions, the ascorbate oxidation that was triggered by three different endophytes in this research may be part of a MAMP-like (microbe-associated molecular patterns) induced response that includes the oxidative burst and initiates plant immune responses. Earlier studies using plant cell suspension cultures demonstrated that different types of molecules from non-pathogenic rhizobacteria, including endophytes, can elicit an oxidative burst (Gerber et al., 2004; Ortmann et al., 2006; van Loon et al., 2008). The wide variety of bacterial molecules that can cause the oxidative burst strongly suggests why the three different bacterial species we used may trigger an oxidative burst, leading to the same extent of ascorbate oxidation by the three endophytes. de Pinto et al. (2003) showed that two different exopolysaccharides (EPS) produced by two different pathogenic bacterial species caused an increase in ascorbate oxidation in tobacco suspension-culture cells, but the total cellular ascorbate pool (AsA + DHA) did not change. The extent of the ascorbate oxidation was similar with the different exopolysaccharides. The EPS-induced ascorbate changes overlapped cellular peroxide increases (de Pinto et al., 2003). Similarly, Ortmann et al. (2006) found that exopolysaccharides from a non-pathogenic rhizobacterium triggered an oxidative burst and peroxide release from plant suspension-culture cells.

The initial contact between endophyte and plant may be at the cell wall/apoplast; therefore, we examined ascorbate oxidase (AO) activity. Ascorbate oxidase is associated with the cell wall/apoplast, but its activity and regulation are not well characterized (Smirnoff, 2000; De Tullio et al., 2013; Foyer et al., 2020). This enzyme catalyzes the oxidation of ascorbate, reducing oxygen to water and monodehydroascorbate (MDHA), which can dismutate to dehydroascorbate (DHA) (Pignocchi and Foyer, 2003; Mellidou and Kanellis, 2024). As is the case with other plants, *Brassica napus* AO activity has not been characterized. Albani et al. (1992) isolated a genomic clone that showed 30% amino acid sequence identity to cucumber and pumpkin ascorbate oxidases upon analysis. Their analysis indicated that this clone may have a different enzymatic activity than other ascorbate oxidases, and it exhibited pollen-specific expression. Batth et al. (2017) used this *Brassica* protein sequence as part of a phylogenetic analysis of AO across diverse genera. The *Brassica* sequence clustered with a rice gene (OsAAO3) and a tobacco gene (NtAAO2), and all three sequences showed significant divergence in protein sequence identity from other ascorbate oxidase proteins (Batth et al., 2017).

**Figure 3:**
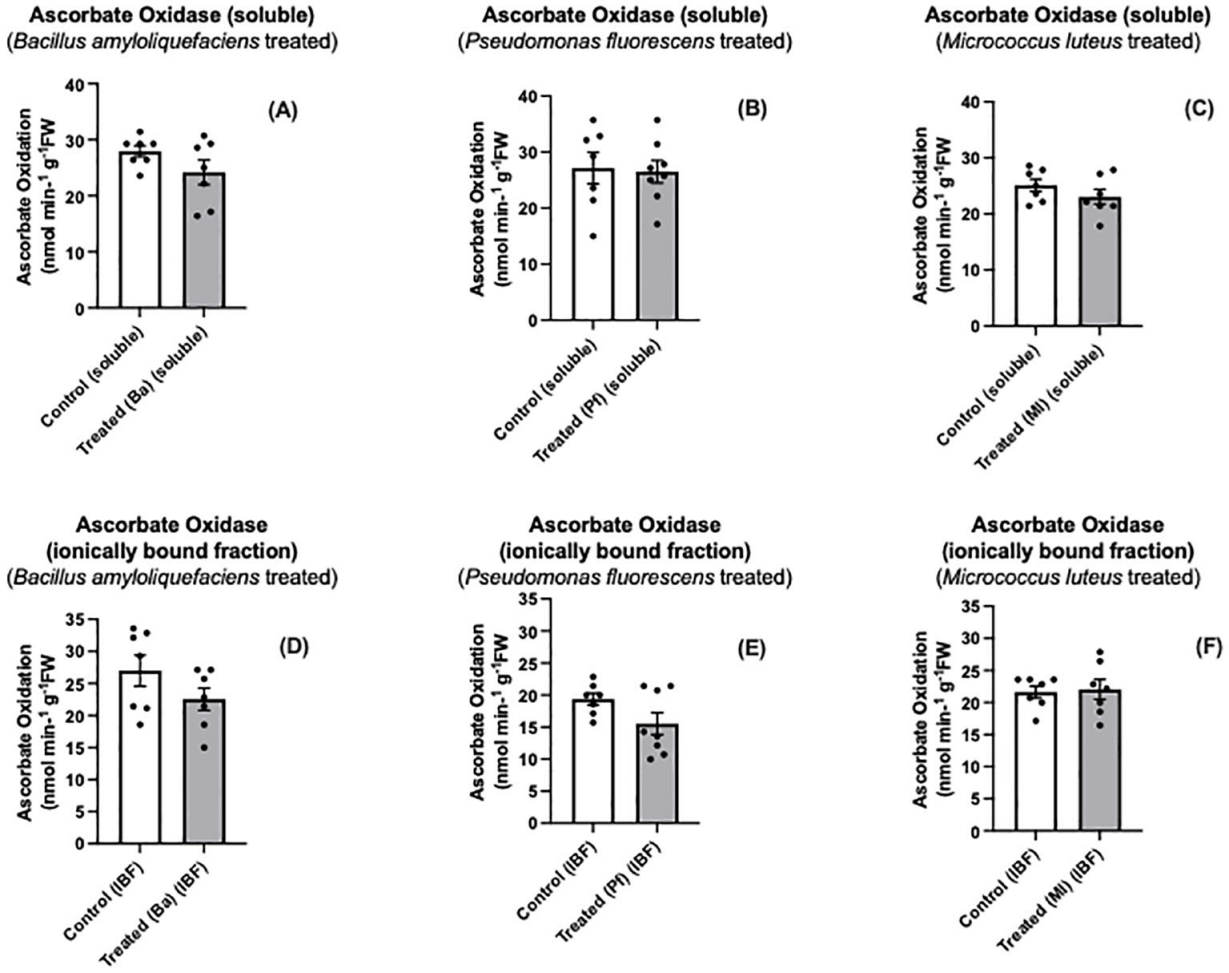
Soluble **(A-C)** and ionically bound **(D-F)** ascorbate oxidase activity in extracts prepared from two-day old *Brassica napus* seedlings treated with different endophytes or untreated controls. Mean ± SEM are indicated (n = 7-8).

**Figure 4:**
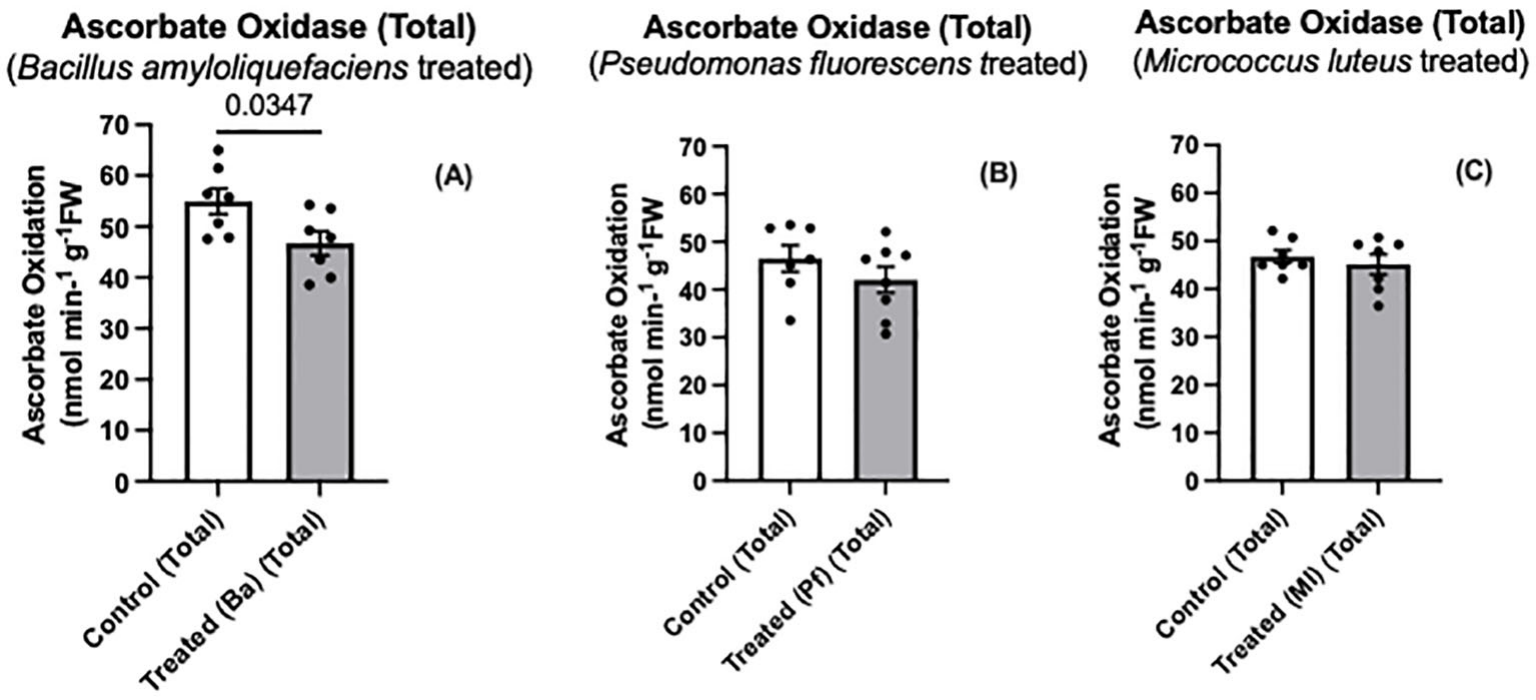
Total ascorbate oxidase activity **(A-C)** in extracts prepared from two-day old *Brassica napus* seedlings treated with different endophytes or untreated controls. Mean ± SEM are indicated (n = 7-8). p-value is indicated when significantly different and was determined using an unpaired two-tailed t-test.

Both Bapb1 and Ml-treated seedlings exhibited reduced soluble AO, while ionically bound (cell wall- associated) AO activity was reduced in *Bacillus* and *Pseudomonas-*treated seedlings compared to control seedlings (**Figures 3A–F**). In terms of total AO activity, Bapb1 treated seedlings had a significantly lower total AO activity as compared to the controls, whereas *Pseudomonas-*treated seedlings, the total activity was slightly reduced (**Figures 4A–C**). Yet, among the endophyte-treated canola seedlings, there was still a level of AO activity that may have been sufficient for the apoplastic ascorbate oxidation that is required for subsequent ROS signaling (**Figures 3**, **4**).

Studies using transgenic and mutant plants with altered AO activity have not provided a clearer understanding of the functional role of the enzyme (Smirnoff, 2018). In an excellent review, Foyer et al. (2020) pointed out that plant lines that highly overexpress or suppress AO activity may result in a plant defense that is less responsive and controlled. Furthermore, a finely regulated AO activity that transiently results in apoplastic ascorbate oxidation may enhance, in conjunction with the oxidative burst, the production of ROS required for effective cell signaling and defense (Foyer et al., 2020).

An example of finely regulated AO activity is the work by Hu et al. (2022) on rice. This study identified an apoplastic effector, MoAo1, as an ascorbate oxidase produced by the rice fungal pathogen *Magnaporthe oryze*. This fungal effector (MoAo1) can bind to and inhibit the enzymatic activity of rice ascorbate oxidase. As part of their detailed study, Hu et al. (2022) isolated a fungal mutant disrupted in the fungal AO gene (ΔMoAo1) that could not inhibit the plant AO activity. In plants where the fungal pathogen AO was active as an effector (wild type) in reducing the plant AO activity, the apoplastic redox status (AsA/AsA+DHA) remained high at 12- and 24-hours post-inoculation. In contrast, in plants inoculated with the pathogen in which the inhibitory fungal AO activity (ΔMoAo1) was disrupted, the plant apoplastic redox status was low 12- and 24-hours post-inoculation because the plant AO was active in oxidizing ascorbate in response to pathogen infection. In the same study, both sets of infected plants showed a decline in symplastic redox status over the course of 36 hours post-inoculation. However, at 48 hours and 72 hours post-inoculation with the wild type fungus that had inhibited rice AO, the symplastic redox status continued to decline. At the same time, plants inoculated with ΔMoAo1 seem to stabilize their symplastic redox status at 48- and 72-hours post-inoculation. By interfering with or reducing the plant’s AO activity, *Magnoporthe* is affecting the apoplastic and symplastic redox states and, in turn, the plant defense response, thus promoting infection. In another study, Wu et al. (2017) identified a rice microRNA that negatively affected rice AO expression. In response to viral infection, this microRNA is sequestered. Both rice studies generally found that reduced plant AO activity promoted viral or fungal infection. When plant AO activity is inhibited, the level of ROS needed for cell signaling and defense is disrupted.

Some endophytes may produce compounds that suppress MAMP-like responses (Millet et al., 2010; Mavrodi et al., 2011). Some of these compounds may be secreted to the extracellular space to modulate the immune response and enhance colonization (Teixeira et al., 2021). Based on our results, both *Bacillus amyloliquefaciens* and *Pseudomonas fluorescens* elicited more of a reduction in AO activity than *Micrococcus luteus* (**Figures 3**, **4**). The differences in altered AO activity dependent on the endophyte may be due to some of these secreted molecules affecting plant AO activity, similar to the rice-*Magnaporthe* interaction.

Peroxide will accumulate intracellularly as part of a pathogen or elicitor-induced response(s) (Nietzel et al., 2019; Hong et al., 2023; Arnaud et al., 2023). One efficient mechanism for regulating peroxide levels is ascorbate peroxidase (APX). APX utilizes ascorbate as an electron donor and reduces peroxide to water and MDHA, which can disproportionate to AsA and DHA (Smirnoff, 2000). We examined cytosolic ascorbate peroxidase activity.

Like ascorbate oxidase, APX has yet to be well characterized in *Brassica napus*. Using the *Arabidopsis* APX genes as a reference, Pan et al. (2022) identified 26 APX homologous genes in *B. napus*. The large number of genes in *B. napus* may be due to the triploid nature of its genome and possible duplications. The expression levels of these different genes varied depending on the abiotic stress (Pan et al., 2022). Their work also suggested possible post-translational regulation of APX activity. However, the regulation of APX may be more complicated. Kaur et al. (2021) reported that *Arabidopsis* cytosolic APX1 not only acted as peroxidase but also could function as a chaperone molecule, depending on its structural form. They also found that post-translational modifications could alter the peroxidase activity.

**Figure 5:**
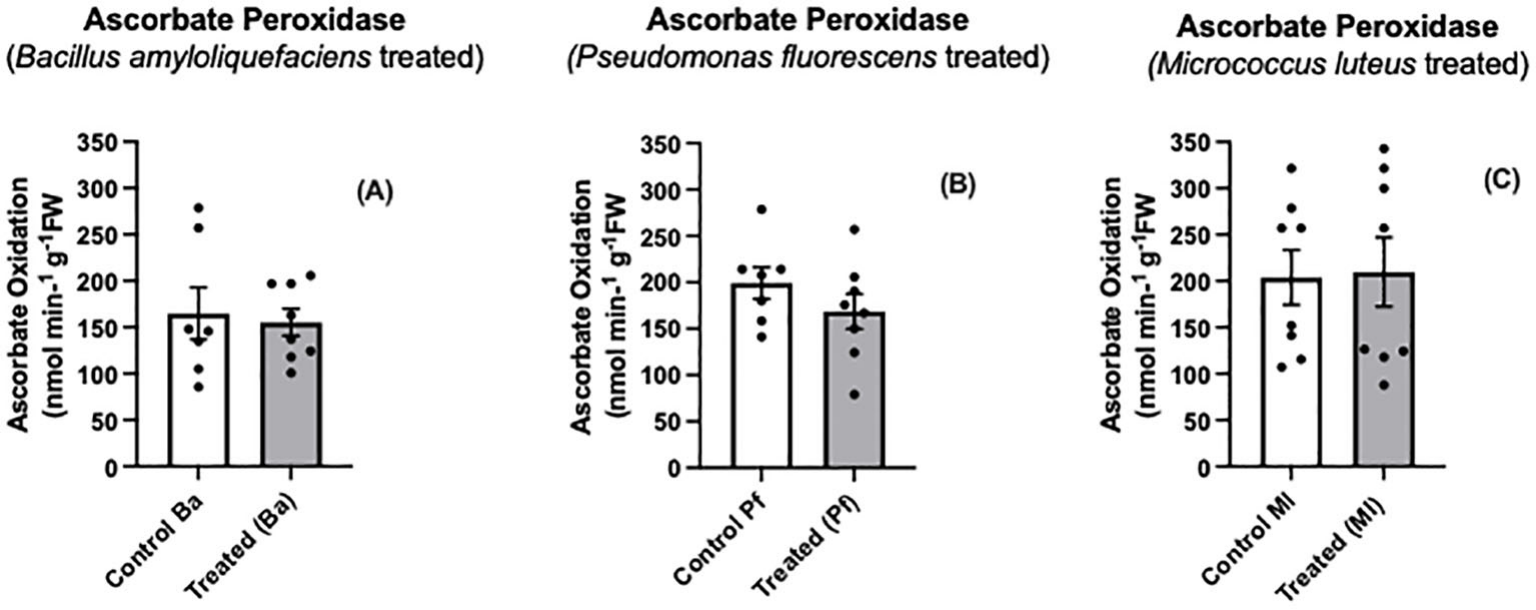
Ascorbate peroxidase activity **(A-C)** in extracts prepared from two-day old *Brassica napus* seedlings treated with different endophytes or untreated controls. Mean ± SEM are indicated (n = 7-8).

We found no differences in ascorbate peroxidase activity between endophyte-treated and control seedlings (**Figures 5A–C**). Using an *Arabidopsis* Col-0 line that expressed a peroxide biosensor (roGFP2-Orp1) in the cytosol, Arnaud et al. (2023) found the bacterial elicitor, flagellin, triggered the intracellular oxidation of roGFP2-Orp1 in leaf discs, but no change in APX activity was detected over the same time interval. Our results do not preclude that other peroxide-scavenging enzymes may be operating.

**Figure 6:**
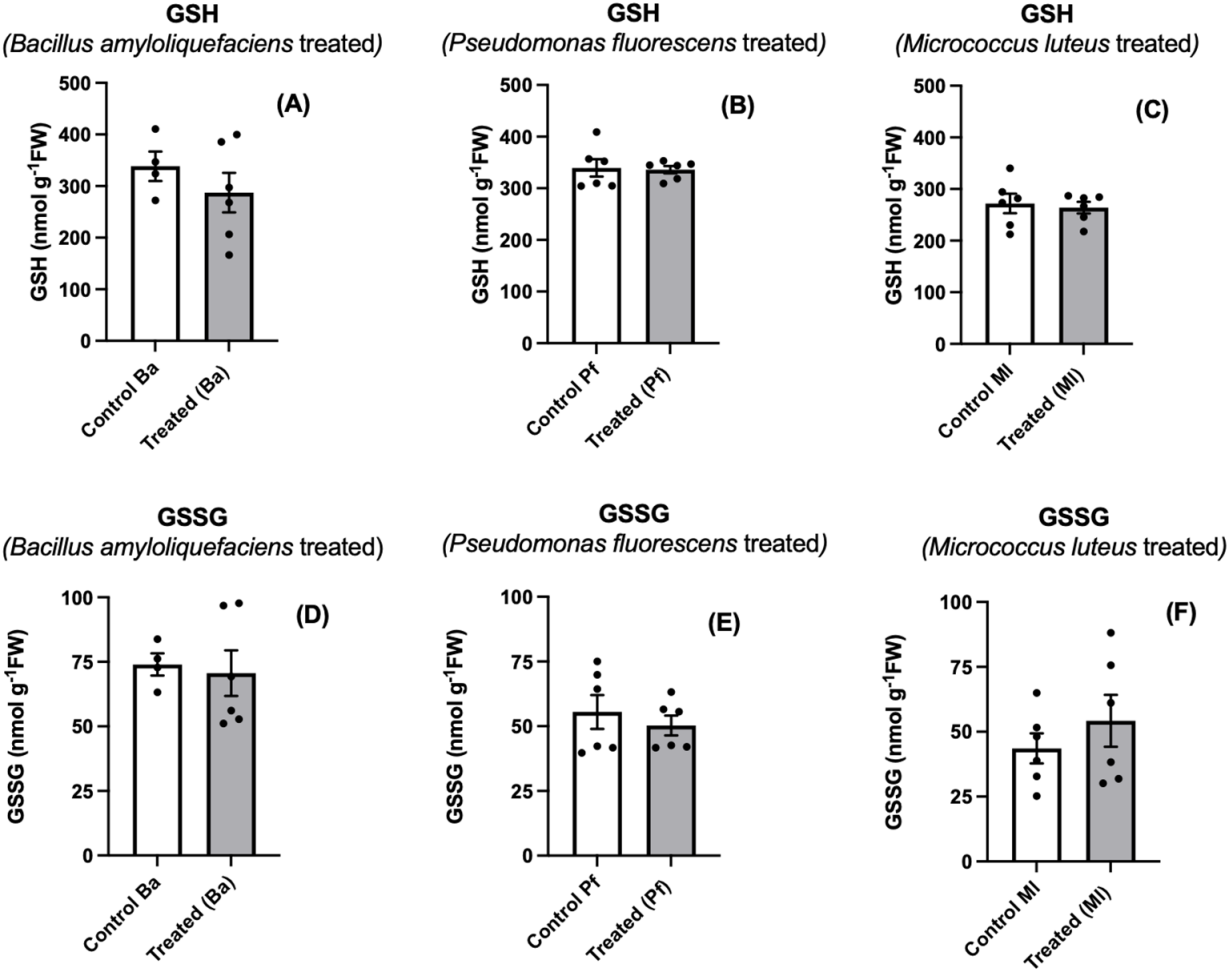
Reduced (GSH) **(A-C)** and oxidized (GSSG) **(D-F)** glutathione in extracts prepared from two-day old *Brassica napus* seedlings treated with different endophytes or untreated controls. Mean ± SEM are indicated (n = 4-6).

As part of our study, we also examined glutathione, another vital antioxidant in plant cells. The role of glutathione in interactions between plants and beneficial microbes is not well defined other than in nitrogen-fixing symbiosis (Frendo et al., 2013). Generally, studies with elicitors or pathogens result in glutathione accumulation, often with a shift toward the oxidized form (Frendo et al., 2013; Noctor et al., 2024). We detected no change in glutathione level or oxidation in endophyte-treated seedlings (**Figures 6A–F**). These results would indicate that, at least at this stage of the plant-endophyte interaction, glutathione is not active in ascorbate regeneration nor is it oxidized by ROS (Foyer and Noctor, 2011).

In summary, ascorbate not only is a major antioxidant in regulating ROS levels, but it also acts as a cofactor in the synthesis of various phytohormones (Smirnoff, 2018; De Tullio, 2020; Xiao et al., 2021). Ascorbate and ROS can interact with plant hormones to affect plant growth and development (Ye et al., 2012; Singh et al., 2024). Such endogenous plant interactions, in addition to endophytic activity such as ACC deaminase and auxin production, may contribute to the early seedling growth effects that we found when seeds were treated with endophytes.

Ascorbate can also affect plant defense pathway activation (Pavet et al., 2005; Mukherjee et al., 2010; Foyer et al., 2020). It is generally thought that endophytes, like pathogens, need to evade or manipulate plant defenses to enable colonization and increase in number (Zamioudis and Pieterse, 2012; Liu et al., 2017). However, endophytes may possibly initiate a moderate plant response relative to pathogens. A moderate plant response would be consistent with some of our results, such as no changes in glutathione or APX activity. The moderate reduction in plant AO activity that we found mimics the inhibitory effects of pathogens on AO activity (Hu et al., 2022). van Loon et al. (2008) found that specific rhizobacterial elicitors triggered the oxidative burst but did not result in cell death overtime in tobacco suspension cells. In contrast, 50% of the cells treated with the pathogenic fungal elicitor cryptogein resulted in cell death (van Loon et al., 2008). Induced systemic resistance (ISR) in *Arabidopsis* by the endophyte *P. fluorescens* WCS417r is thought to involve a “moderate or localized stimulation of the jasmonate and ethylene response” that was not sufficient for gene activation, including defense genes (Pieterse et al., 1998).

Finally, perhaps the ascorbate oxidation that we found is the key event in the initial interaction between endophytes and plants and may lead to the priming of the plant for further response to environmental stress. In terms of priming, some of the molecular components thought to be required are mitogen-activated protein kinases (MAPKs), which are involved with signal transduction and chromatin modifications as they affect gene regulation (Conrath, 2011; Conrath et al., 2015). ROS and ascorbate oxidation can affect MAPKs (Kovtun et al., 2000; Pignocchi and Foyer, 2003; Pignocchi et al., 2006; Pitzschke and Hirt, 2006). Also, several recent reviews have indicated that ascorbate may affect epigenetic changes in plants, although this has not been demonstrated in plants at this time (Bellini and De Tullio, 2019; Xiao et al., 2021; Foyer and Kunert, 2024). Therefore, it is quite possible that ascorbate oxidation may be part of the priming mechanism in plants.

## Data Availability

The original contributions presented in the study are included in the article/supplementary material. Further inquiries can be directed to the corresponding author/s.
